# Bibliometric analysis of research on the trends in autophagy

**DOI:** 10.7717/peerj.7103

**Published:** 2019-06-05

**Authors:** Ting Hong, Xinzhe Feng, Wenwen Tong, Weidong Xu

**Affiliations:** 1 Department of Endocrinology, Drum Tower Hospital, Nanjing University Medical School, Nanjing, Jiangsu, P.R. China; 2 Department of Orthopedics, Changhai Hospital, Second Military Medical University, Shanghai, Shanghai, P.R. China

**Keywords:** Citation, H-index, Autophagy, Bibliometric, VOS viewer

## Abstract

**Background:**

Autophagy is an important mechanism to maintain homeostasis in cells. It has been linked with ageing and many currently incurable diseases, including heart disease, cancer, myopathies, neurodegeneration, and diabetes. Autophagy research is very important for identifying better treatments. This study aimed to explore the hotspots of autophagy research published from different countries, organizations, and authors.

**Methods:**

Between 1962 and 2018, articles published about autophagy were identified in the Web of Science database. The total and annual number of articles, citations, impact factor, Hirsch (H)-index, number of article citations, productive authors, and involved journals were collected for quantitative and qualitative comparisons.

**Results:**

From 1962 to 2018, 18,811 autophagy-related articles written in English were published. Most were from China (6,731). The United States dominated in citation frequency (391,030) and h-index (264). Among related journals, *Autophagy* published the most articles (1,388), followed by *Plos One* (585) and *Oncotarget* (392). Daniel Klionsky was the most productive author, with 171 publications. The article “LC3, a mammalian homologue of yeast Apg8p, is localized in autophagosome membranes after processing” was cited most frequently. The top-ranked keyword was “degradation” of macroautophagy.

**Conclusions:**

Publication of articles about autophagy has increased notably from 1962 to 2018, and has increased annually. The general quality of publications from China is still in need of improvement. Autophagy research has shifted gradually from basic studies to clinical studies in recent years.

## Introduction

Autophagy is a highly conserved mechanism of the controlled digestion of damaged organelles that is a common phenomenon in eukaryotes (Ciccia & Haroon, 2016). It was first reported in 1962 in an electron microscopy study of human liver cells (Ashford & Porter, 1962). Based on the differences in mechanisms and functions, autophagy is usually classified into three types: macroautophagy, microautophagy, and chaperone-medicated autophagy. Autophagy is closely related to the growth and differentiation of organisms. It can help cells to clear abnormally misfolded or aggregated proteins, and provides materials for the construction of new organelles to maintain cellular homeostasis (Rusten et al., 2008). Autophagy also plays an important role in resisting bacterial invasion. Autophagic dysfunction is associated with a wide range of human diseases, such as cancer, neurodegenerative diseases, inflammation, and immune system diseases (Cheung & Ip, 2011; Levine, Mizushima & Virgin, 2011). Reflecting this importance, autophagy research has dramatically risen in recent years. A better understanding of autophagy will clarify the pathogenesis of some presently incurable diseases, which could ultimately lead to cures. The importance of autophagy was recognized by the awarding of the 2016 Nobel Prize in Physiology or Medicine for research in autophagy (Levine & Klionsky, 2017).

Bibliometric analysis is an important tool that can help assess new trends in current scientific research (Pu, Lyu & Su, 2016). This technology allows people to quickly acquire information concerning the scientific output of individuals, institutions, or countries using relevant parameters that include quantity, impact factor (IF), and citation of published papers over time (Zyoud et al., 2016). It is a well-recognized technique of systematic analysis (van Raan, 2005) and has played a fundamental role in policy making and establishing clinical guidelines(Wang et al., 2016). Bibliometric analysis is common in a variety of different disciplines, such as diabetes (Geaney et al., 2015), ophthalmic diseases (Boudry, Baudouin & Mouriaux, 2018), organ transplantation (Pu et al., 2017), toxicology research (Sweileh et al., 2014), and molecular biology (Miao et al., 2017).

This study used bibliometric tools to analyse autophagy-related articles retrieved from several databases. The aim was to provide a retrospective and contemporary view of the mainstream research on autophagy globally from 1962 to 2018.

## Materials and Methods

The online literature search was done on March 25, 2019 using the Web of Science (Thomas Reuters Company) citation indexing service that allowed access to the Essential Science Indicators (ESI) and Science Citation Index Expanded databases. Articles and reviews were included in the analysis, because they generally accounted for the majority of document types that also included complete research ideas and results. Terms used during the search were: TI = autophag* AND publishing year = (1962–2018) AND Language = English. Data entry and collecting were verified by two authors (Feng XZ and Tong WW). The txt data downloaded from the Web of Science were imported into Microsoft Excel 2013 and VOSviewer. The data were analysed quantitatively and qualitatively.

The Web of Science allows the analyses of many publication characteristics, including countries or regions, institutions, publication time, authors, citation frequency, ESI top papers, and the Hirsch (H)-index. The latter is often applied to evaluate a scholar’s academic level more scientifically. The definition of the index is that a scholar with an index of H has published H papers, each of which has been cited in other papers at least H times (Mahaisavariya, 2010). It is often used to measure both the productivity and citation impact of the publications of a scholar (Jacsó, 2012).

Microsoft Excel 2013 was used to chart the trend of publications according to the Web of Science data. The equation *f*(*x*) = *ax*^3^ + *bx*^2^ + *cx* + *d* was used to predict the future trend of papers in this field. The publication year is denoted by *x* and *f*(*x*) is the total number of publications in any one year. The plotted data can also be analysed to understand the difference of publication outputs between different countries or institutions through column diagrams or line charts.

The VOSviewer software tool is used to construct and visualize bibliometric networks, which include journals, researchers, or individual publications. The constructs can be based on citations, bibliographic coupling, co-citation, or co-authorship relations (Khalil & Gotway Crawford, 2015). In addition, VOSviewer offers text mining functionality that can be used to perform and visualize co-occurrence networks of vital terms extracted from a body of scientific literature (van Eck & Waltman, 2010). The GoPubMed website (http://www.gopubmed.org/web/gopubmed/) was harnessed to count the data of relative research interest.

## Results

### Publication outputs and growth prediction

**Figure 1:**
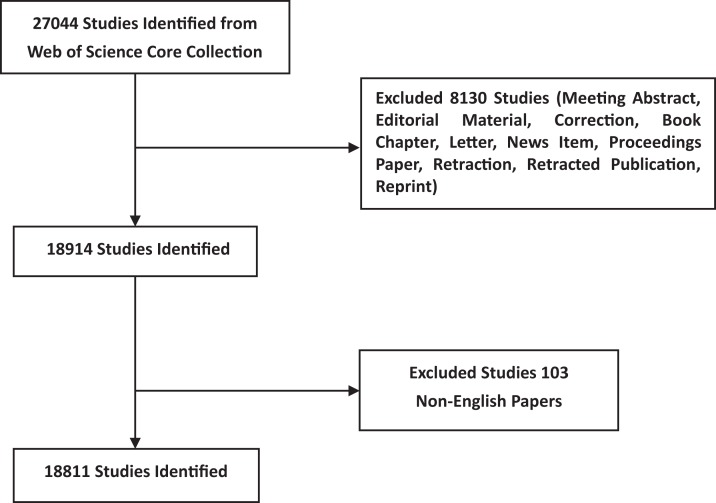
Flow diagram of identification of autophagy studies.

**Figure 2:**
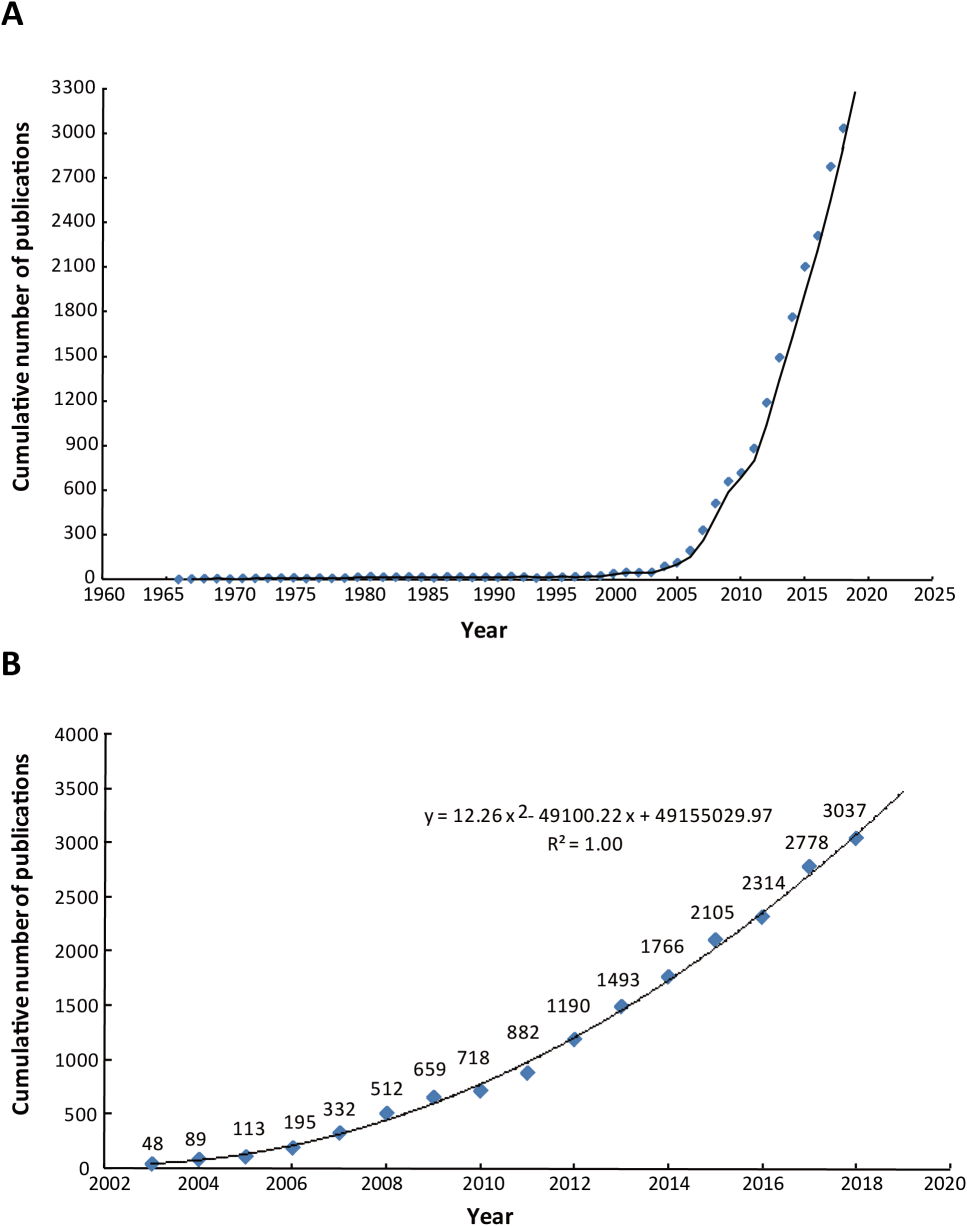
Trend of changes in number of publications worldwide. (A) The trend of changes from 1966 to 2018. (B) The trend of changes from 2003 to 2018.

**Figure 3:**
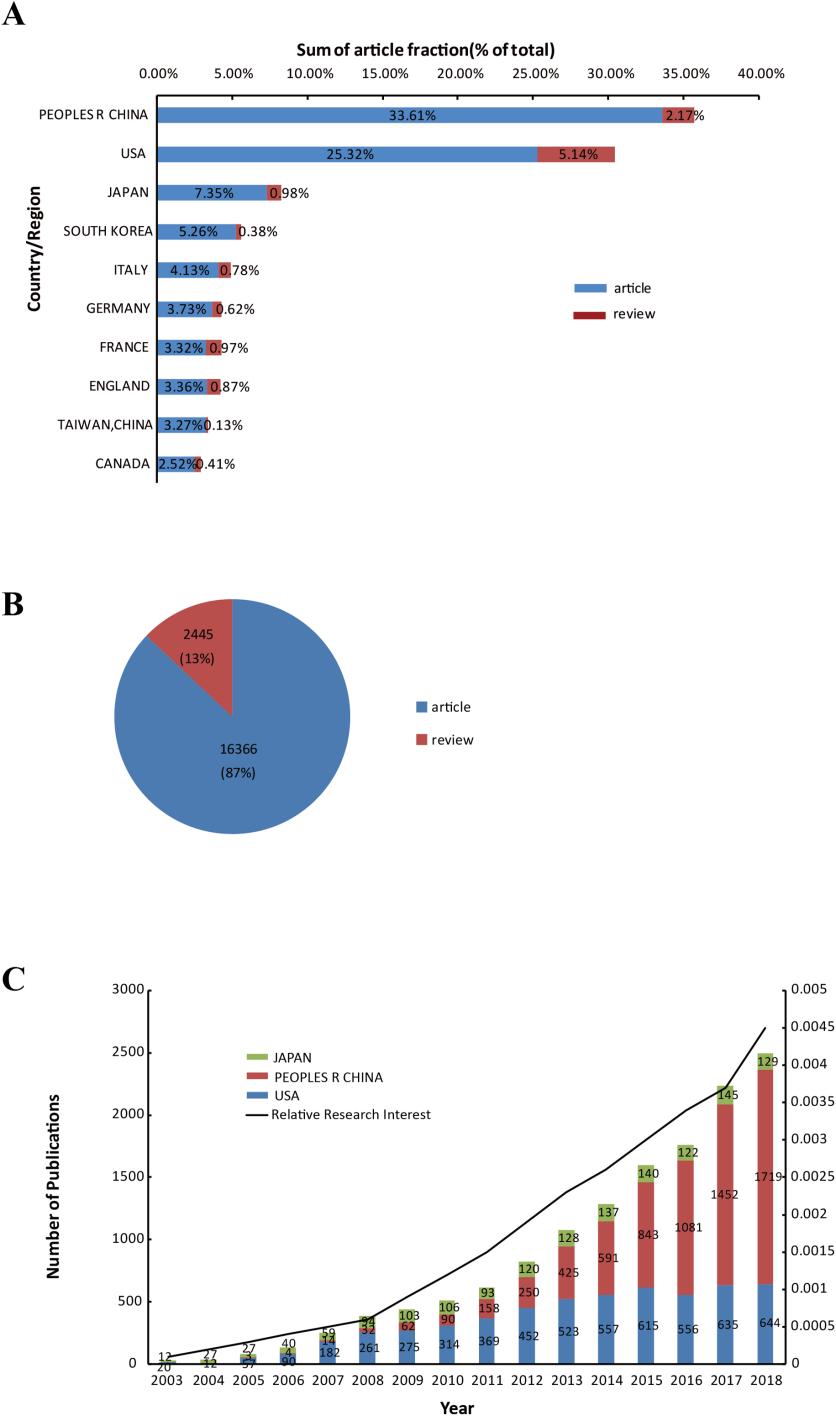
Contributing characteristics of autophagy research. (A) Sum of autophagy research-related article fractions (% of research from all regions) from the top 10 countries or regions. (B) The number and proportion of articles and reviews on autophagy globally. (C) Number of publications from the top three countries and the research interests.

A total of 18,811 articles met the search criteria from 1962 to the end of 2018 (Fig. 1; Fig. S1). The first paper we were able to find in the Web of Science with the word “autophagy” in the title was published in 1966 (Kent et al., 1966). The number of such articles rose dramatically from 1 in 1966 to 3,037 in 2018. The number of annual publications has risen rapidly in the last 15 years (Fig. 2), with an annual increase in the number of articles (Fig. 3C; Fig. S2).

Substantial progress in the molecular study of autophagy has taken place during only the past 15 years (Yang & Klionsky, 2010), so we selected data from the last 15 years to depict the model fitting curve in Fig. 2. As shown in Fig. 2B, the model fitting curve calculated by the growth of autophagy publications indicated a significant correlation (*R*^2^ = 1.00) between the cumulative number of publications and publication year. According to the statistics of the past 15 years, we forecast that there will be approximately 3,500 autophagy-related articles published in 2019.

### Countries/regions contributing to global publications and growing trends

The 18,811 publications focusing on autophagy research were contributed by 100 countries/territories (Dataset S1). Figure 3A provides a bar chart of the top 10 countries, based on the percentage of publications. China published the most original articles (6,322, 33.61%), followed by the United States (4,762, 25.32%) and Japan (1,383, 7.35%). The US published the most review articles (967, 5.14%). The number of original articles (16,366, 87%) was approximately seven times the number of review articles (2,445, 13%) in all countries (Fig. 3B). The results of Fig. 3C demonstrate that articles from China soared from zero in 2003 to 1,719 in 2018. In addition, the most articles per year after 2014 were published by researchers from China. The number of publications from the US increased from 20 in 2003 to 644 in 2018. The number of publications from China and the US were comparable in 2014. The number of publications from Japan increased gradually from 12 in 2003 to 129 in 2018 (Fig. 3C).

### Distribution of autophagy journals

Table 1 lists the top 10 most productive journals that published articles on autophagy research. The corresponding values (year 2017) of their IFs in the Web of Science database are also included. Journals from the US and the United Kingdom occupied the top seven rankings. The most prolific journal has been the US publication *Autophagy* with 1,388 articles (7.379% of the total), followed by *Plos One* (585, 3.11%) and *Oncotarget* (392, 2.084%), which are also US journals.

**Table 1:** Top 10 journals that published articles on autophagy research.

Rank	Journal title	Country	Count	Percent	IF 2017
1	*Autophagy*	USA	1,388	7.379	11.100
2	*Plos One*	USA	585	3.110	2.766
3	*Oncotarget*	USA	392	2.084	
4	*Journal of Biological Chemistry*	USA	390	2.073	4.01
5	*Biochemical and Biophysical Research Communications*	USA	385	2.047	2.559
6	*Scientific Reports*	UK	372	1.978	4.122
7	*Cell Death & Disease*	UK	344	1.829	5.638
8	*Molecular Medicine Reports*	Greece	214	1.138	1.922
9	*Proceedings of the National Academy of Sciences of the United States of America*	USA	169	0.898	9.504
10	*Cell Death and Differentiation*	UK	165	0.877	8.0

### Distribution of authors

The 10 most prolific authors contributed a total of 1,201 articles relating to autophagy, accounting for 6.38% of all published literature relating to the field. Daniel J. Klionsky from the University of Michigan in the US has published the most articles (171), followed by Mizushima Noboru of Tokyo Medical Dental University (Japan) with 140 publications and Ohsumi Yoshinori of the Tokyo Institute of Technology (Japan) with 133 publications. Dr. Klionsky has been the only person to publish more than 150 articles on autophagy from 1962 to 2018. Authors from the US and Japan make up the majority of the top 10 authors rankings. Of the top 10 authors, six are from universities and four are from institutes (Table 2). Mizushima Noboru, Daniel Klionsky, and Ohsumi Yoshinori are the top three concerning H-index (Table S1).

**Table 2:** Top 10 authors with the most publications related to autophagy research.

Author	Number of papers	Country	Affiliations
Klionsky Daniel J	171	USA	University of Michigan
Mizushima Noboru	140	Japan	The University of Tokyo
Ohsumi Yoshinori	133	Japan	Tokyo Institute of Technology
Yoshimori Tamotsu	120	Japan	Osaka University
Cuervo Ana Maria	116	USA	Albert Einstein College of Medicine
Levine Beth	111	USA	University of Texas Southwestern Medical Center
Kroemer Guido	108	France	Institut National de la Sante et de la Recherche Medicale (Inserm)
Codogno Patrice	102	France	Institut National de la Sante et de la Recherche Medicale (Inserm)
Rubinsztein David C	102	UK	University of Cambridge
Komatsu Masaaki	98	Japan	Juntendo University

### Citation and H-index analysis

The Web of Science analysis revealed that articles related to autophagy had been cited 793,192 times from 1962 to 2018 (650,834 times without self-citations). Each article was cited 42.17 times. The top 10 countries ranked by the citation and H-index on autophagy research were determined.

**Figure 4:**
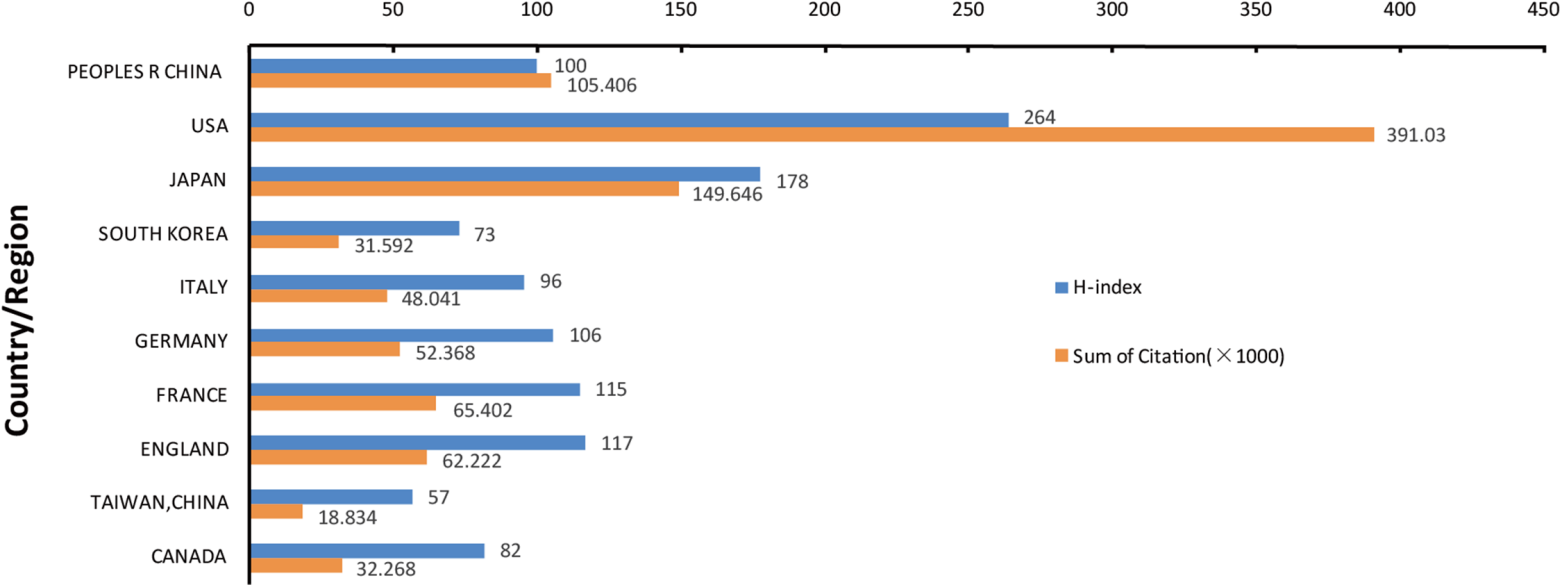
Citation and H-index analysis of the top 10 countries or regions.

The H-index positively correlated with the sum of citations (Fig. 4). The H-index of the US and Japan clearly exceeded the other eight countries. The number of citations of articles from the US was 391,030 (49.3% of the total), followed by Japan (149,646, 18.9% of the total). The H-index was 264 in the US. Japan ranked second (178) and England ranked third (117). The number of publications from China ranked first in the list. However, the citation frequency and H-index ranked third and sixth, respectively.

### Most frequently cited articles

The top 10 cited articles about autophagy are listed in Table 3. The range in the number of citations varied from 2,161 for the article in the 10th position to 4,118 for the leading article. All ten articles were cited more than 2,000 times. Seven of the articles are reviews and the remaining three are original articles. In addition, more than half were published in journals with an IF above 20.

**Table 3:** Top 10 studies with the most citation frequency related to autophagy research.

Title	First author	Journal (IF2017)	Year	Citation frequency	Main conclusion
LC3, a mammalian homologue of yeast Apg8p, is localized in autophagosome membranes after processing	Kabeya, Yukiko	*Embo Journal* (10.557)	2000	4,118	The authors demonstrate that the rat microtubule-associated protein 1 light chain 3 (LC3), a homologue of Apg8p essential for autophagy in yeast, is associated with the autophagosome membranes after processing.
Autophagy in the pathogenesis of disease	Levine, Beth	*Cell* (31.398)	2008	3,672	This review summarizes recent advances in understanding the physiological functions of autophagy and its possible roles in the causation and prevention of human diseases.
Autophagy fights disease through cellular self-digestion	Mizushima, Noboru	*Nature* (41.577)	2008	3,549	This review describes the main mechanisms of autophagy and its role in disease.
Development by self-digestion: Molecular mechanisms and biological functions of autophagy	Mizushima, Noboru	*Developmental Cell* (9.616)	2004	2,460	In this review, the authors focus on macroautophagy, an evolutionarily served process that occurs in virtually all eukaryotic cells, ranging from yeast to mammals.
Methods in Mammalian Autophagy Research	Mizushima, Noboru	*Cell* (31.398)	2010	2,287	In this Primer, the authors discuss methods to monitor autophagy and to modulate autophagic activity, with a primary focus on mammalian macroautophagy.
Autophagy as a regulated pathway of cellular degradation	Klionsky, Daniel J	*Science* (41.058)	2000	2,230	This review limits the discussion to macroautophagy, the major inducible pathway for general turnover of cytoplasmic components.
Suppression of basal autophagy in neural cells causes neurodegenerative disease in mice	Hara, Taichi	*Nature* (41.577)	2006	2,220	In this article, the authors report that loss of autophagy causes neurodegeneration even in the absence of any diseaseassociated mutant proteins.
AMPK and mTOR regulate autophagy through direct phosphorylation of Ulk1	Kim, Joungmok	*Nature Cell Biology* (19.064)	2011	2,212	This article reveals a signalling mechanism for Ulk1 regulation and autophagy induction in response to nutrient signalling.
Guidelines for the use and interpretation of assays for monitoring autophagy	Klionsky, Daniel J	*Autophagy* (11.1)	2012	2,177	A set of guidelines are presented for the selection and interpretation of methods for use by investigators, as well as for reviewers who need to provide realistic and reasonable critiques of papers that are focused on these processes.
Bcl-2 antiapoptotic proteins inhibit Beclin 1-dependent autophagy	Pattingre, Sophie	*Cell* (31.398)	2005	2,161	This article shows that wild-type Bcl-2 antiapoptotic proteins, but not Beclin 1 binding defective mutants of Bcl-2, inhibit Beclin 1-dependent autophagy in yeast and mammalian cells and that cardiac Bcl-2 transgenic expression inhibits autophagy in mouse heart muscle.

Among these 18,811 articles, the three most frequently cited are: “LC3, a mammalian homologue of yeast Apg8p, is localized in autophagosome membranes after processing” by Yukiko Kabeya, published in 2000 in *EMBO Journal* (4,118 citations, IF: 10.557); “Autophagy in the pathogenesis of disease” by Beth Levine, published in 2008 in Cell (3,672 citations, IF: 31.398); and “Autophagy fights disease through cellular self-digestion” by Noboru Mizushima, published in 2008 in Nature (3,549 citations, IF: 41.577). Overall, articles from the US and Japan have been the most highly cited. Almost all the top 10 cited articles are from these two countries.

### Hotspots of studies on autophagy

**Figure 5:**
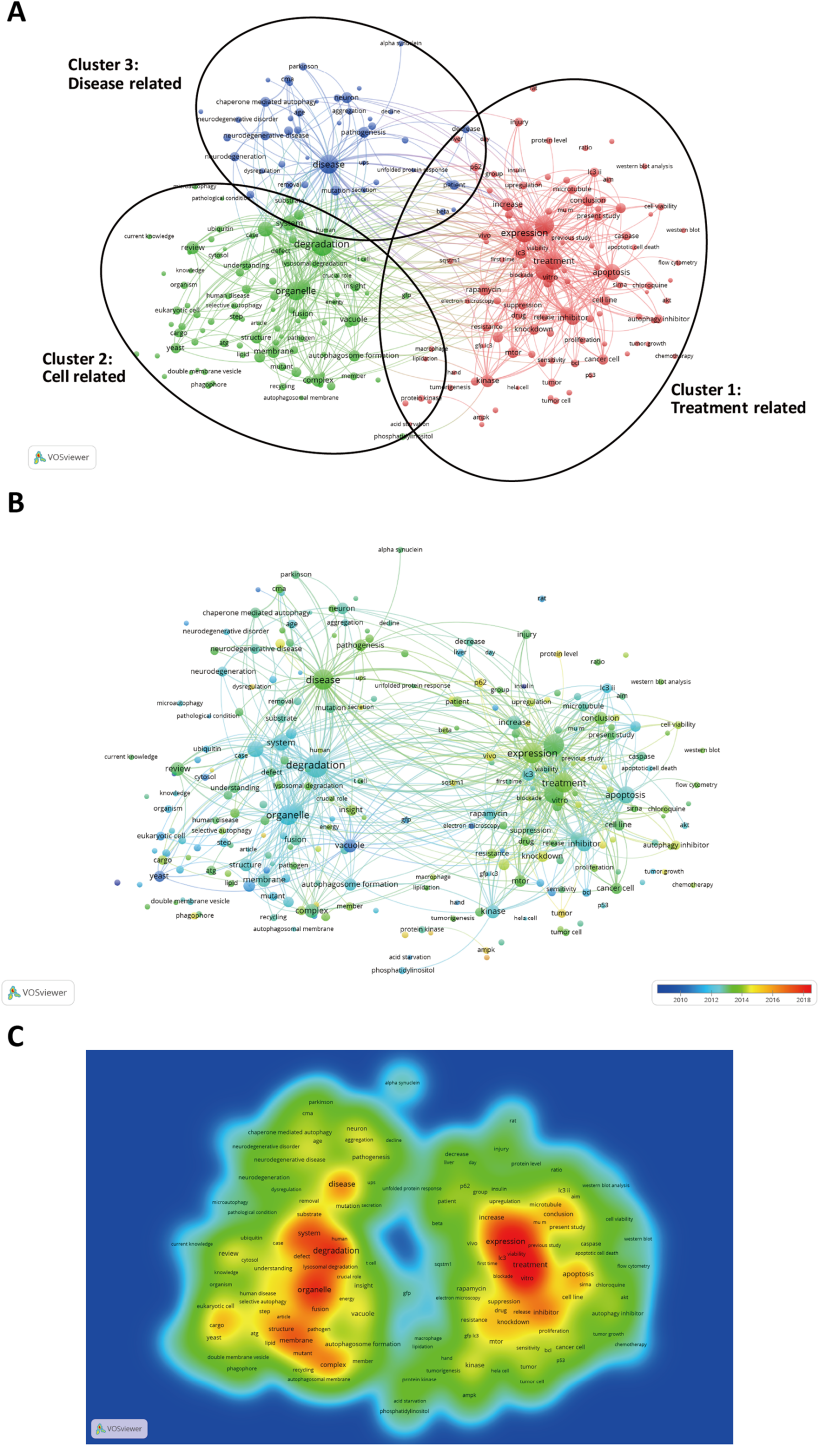
The analysis of keywords. (A) Mapping on keywords of autophagy. The keywords were divided into three clusters according to different colours generated by default: Treatment-related study (right in red), Cell-related study (left in green), Disease-related study (up in blue). A large circle indicates that the keyword appears at a high frequency. (B) Distribution of keywords according to when they appeared for the average number of times. Keywords in blue are presented earlier than those in yellow or red. Two terms are defined to co-occur if they both occur on the same line in the corpus file. In general, the smaller the distance between two terms, the larger the number of co-occurrences of the terms. (C) Mapping on keywords of autophagy. The words in central red area were used most frequently.

Keywords of all the 18,811 original articles and reviews were evaluated using VOSviewer. The three types of autophagy are completely different, so we separately searched for publications concerning macroautophagy, microautophagy, and chaperone-mediated autophagy. The article numbers were 1,552, 99, and 722, respectively. The content of the remaining articles was indistinguishable. Macroautophagy is the main form of autophagy and has been studied most thoroughly. As shown in Fig. 5, the 248 keywords (defined as being used more than 20 times within titles and abstracts in all the articles) with 21,763 links were classified into three clusters: “Treatment related” (Amaravadi et al., 2011), “Cell related” (Klionsky & Emr, 2000), and “Disease related” (Levine & Kroemer, 2008; Mizushima et al., 2008) (Fig. 5A). In the “Treatment related” cluster, the primary keywords were expression (374 times), effect (373 times), and treatment (342 times). For the “Cell related” cluster, the primary keywords were degradation (480 times), organelle (350 times), and system (237 times). For the “Disease related” cluster, the primary keywords were disease (375 times), pathogenesis (122 times), and neuron (119 times).

Figure 5B depicts an overlay visualization, in which the VOSviewer was used to apply colours to keywords according to the year of publication. The software automatically assembled statistics of the keywords from 2010 to 2018. Keywords with a cool colour appeared early and those with a warm colour appeared later. In the early years of autophagy research, the main topics were degradation (cluster 2, average publishing year (APY) of keywords = 2012.68) and organelle (cluster 2, APY = 2012.06). With time, the keywords concentrated in the “Treatment related” and “Disease related” clusters. In the “Treatment related” cluster, “becn1” (cluster 1, APY = 2015.81) was a keyword 32 times. In the “Disease related” cluster, “dysregulation” (cluster 3, APY = 2015.00) was a keyword 36 times. The most important keywords were located in two regions (Fig. 5C). The left region contains two representative keywords: disease (247 links, 375 occurrences) and degradation (247 links, 480 occurrences). The right region contains two representative keywords: expression (245 links, 374 occurrences) and treatment (245 links, 342 occurrences). Autophagy was mainly related to cell biology, biochemistry molecular biology, and oncology (Fig. S3).

The keywords of microautophagy were obviously less than keywords of chaperone-mediated autophagy and macroautophagy. More keywords were used for chaperone-related autophagy compared to microautophagy, but their use grew with time (Figs. S4 and S5).

## Discussion

### Trend of autophagy research

The quantity of global publications on autophagy has increased rapidly year-by-year from 2003 to 2018. Since 2012, the total number of articles on autophagy has increased rapidly. It can be speculated that there was a big breakthrough in autophagy research around 2012. Graphing the data highlights that autophagy research is a hotspot at the present time. Recent times have been a golden period of the development of autophagy research. China is by far the most productive country, followed by the US and Japan. Although these countries have published many papers, the indicators of H-index and sum of citations are different from each other.

The US ranks first among all countries in H-index and total number of publications. This suggests the high-quality and numbers of documents from the US. There are several potential reasons. The GDP of the US is the highest globally. Thus, the US is able to fund the necessary scientific research. Secondly, the finding that three of the top 10 researchers are Americans, second only to Japan, is consistent with the view that American scientists have conducted in-depth studies of autophagy for a long time, with the discoveries laying a solid foundation for later research (Klionsky & Emr, 2000; Liang et al., 1998). Moreover, the many excellent universities and research institutes in the US run ahead of other countries in autophagy research.

Although Japan ranks third among the top 10 productive countries in terms of the number of articles published, the H-index, quantity of productive authors, and highly cited articles on autophagy are all far ahead of most countries. Additionally, the Japanese scientist Yoshinori Ohsumi won the 2016 Nobel Prize Physiology or Medicine because of his contribution to research on autophagy. In the 1990s, Dr. Ohsumi discovered the process of autophagy and its important role in yeast. His ground-breaking article published in the *Journal of Cell Biology* reported for the first time that autophagy occurs in yeast under conditions of starvation, and that autophagy is essential for yeast survival (Takeshige et al., 1992). Subsequently, his team searched for the genes necessary for yeast survival and discovered several key genes involved in autophagy regulation. This important work was published in the FEBS Letters in 1993 (Tsukada & Ohsumi, 1993). In 1997, Dr. Ohsumi’s group cloned the first autophagy gene, *apg1*, in yeast (Matsuura et al., 1997). The researches headed by Dr. Ohsumi group are regarded as landmarks of the field of autophagy. Research by Dr. Ohsumi and colleagues, and others has positioned Japan at the forefront of autophagy research.

China is the only developing country of the top 10 productive countries. China experienced a meteoric rise in productivity from only zero articles in 2004 to 1,719 (ranked first that year) in 2018. Since 2014, China has been publishing more articles each year than any other country. In 2004, there were no articles published from China, with the US and Japan respectively publishing 12 and 27 articles. These findings provide more evidence that research on autophagy in Japan and the US began sooner than elsewhere. The US, Japan, England, France, and Germany published articles with a higher H-index than China.

Autophagy plays an important role in nervous system diseases and tumours (Liang & Sigrist, 2017; White, 2015). Along with an increasingly ageing global society, the number of people suffering from nervous system diseases and cancer in China has been increasing year by year. Studies on the pathogenesis of these diseases have attracted more attention than the mechanism of autophagy. Additionally, with the economic improvement, more Chinese universities/institutes will acquire funds to support research. China lacks talented researchers and has started autophagy research later than other countries. This is reflected in the lack of inclusion of Chinese scholars and articles in Tables 2 and 3. China still has a long way to go to reach the scientific research level of developed countries.

The journal *Autophagy* was by far the most pre-eminent journal, with 1,388 articles, followed by *Plos One*, *Oncotarget*, and *Journal of Biological Chemistry*. They are expected to be the sources of many more articles about autophagy.

With respect to authors and publications, highly cited articles were mainly written by the 10 most prolific authors. Their research focused on the mechanisms of autophagy.

### Research focused on autophagy

Autophagy-related documents that were cited the most will provide the fundamental basis for further studies. Additionally, they provide a reasonable prediction of research frontiers. According to the main conclusions of the top two highly cited reviews, the current focus of research is the link between autophagy and disease. Many of the documents that have been cited most often were published in 2010. This likely reflects the advances being made on the mechanism of autophagy at that time. Reading articles about autophagy published at that time could meaningfully inform future research.

Among the 10 most productive journals, *Autophagy* is the only journal that specially publishes articles on all aspects of autophagic processes. Most of the other journals almost logically belong to the “Multidisciplinary Sciences” JCR category, confirming the important role that Autophagy plays in the dissemination of information of autophagy research in the life sciences community.

Autophagy research has shifted gradually from basic studies to clinical studies in recent years. Initially, autophagy in yeast cells was the main focus (Klionsky & Emr, 2000; Matsuura et al., 1997). With further research, the involvement of autophagy was identified in many diseases, especially nervous system diseases (Liang, 2019; Nikoletopoulou & Tavernarakis, 2018), cancer (Galluzzi et al., 2015), and autoimmune diseases (Gianchecchi, Delfino & Fierabracci, 2014). This is reflected in Fig. 5B.

## Conclusions

This is the first bibliometric study assessing the worldwide productivity in the field of autophagy research. Publications on autophagy will continue to grow rapidly. The United States has been the most productive country as measured by total citations and H-index. Among related journals, *Autophagy* has published the largest number of articles in autophagy research. Daniel Klionsky, Noboru Mizushima, and Ohsumi Yoshinori may be good candidates for collaborative research in this field based on the H-index and citation frequency of their publications. The article“LC3, a mammalian homologue of yeast Apg8p, is localized in autophagosome membranes after processing” is the most cited publication. All publications can be divided into three clusters. The “Treatment related”, “Cell related”, and “Disease related” clusters may be the latest hotspots of study, and research in these areas may drive the field of autophagy in the next few years.

## Supplemental Information

10.7717/peerj.7103/supp-1 Supplemental Information 1 Countries or territories involved in autophagy research that were extracted from the Web of Science Core Collection.

10.7717/peerj.7103/supp-2 Supplemental Information 2 Raw data performed by VOS viewer.

10.7717/peerj.7103/supp-3 Supplemental Information 3 The H-index and citation frequency of top 10 productive authors on autophagy research.

10.7717/peerj.7103/supp-4 Supplemental Information 4 Search queries used in Web of Science Core Collection.

10.7717/peerj.7103/supp-5 Supplemental Information 5 The data of relative research interest.

10.7717/peerj.7103/supp-6 Supplemental Information 6 Search queries used in Web of Science Core Collection.

10.7717/peerj.7103/supp-7 Supplemental Information 7 Analysis of keywords of chaperone-medicated autophagy by Vosviewer.

10.7717/peerj.7103/supp-8 Supplemental Information 8 Analysis of keywords of microautophagy by Vosviewer.

## Additional Information and Declarations

### Data Availability

The following information was supplied regarding data availability: The raw measurements are available in the Supplemental Files.
