# Supplementary figures and images for: Bibliometric analysis of research on the trends in autophagy
Source: PeerJ. 2019 Jun 5;7:e7103. doi: 10.7717/peerj.7103 (PMC6556104; doi:10.7717/peerj.7103)

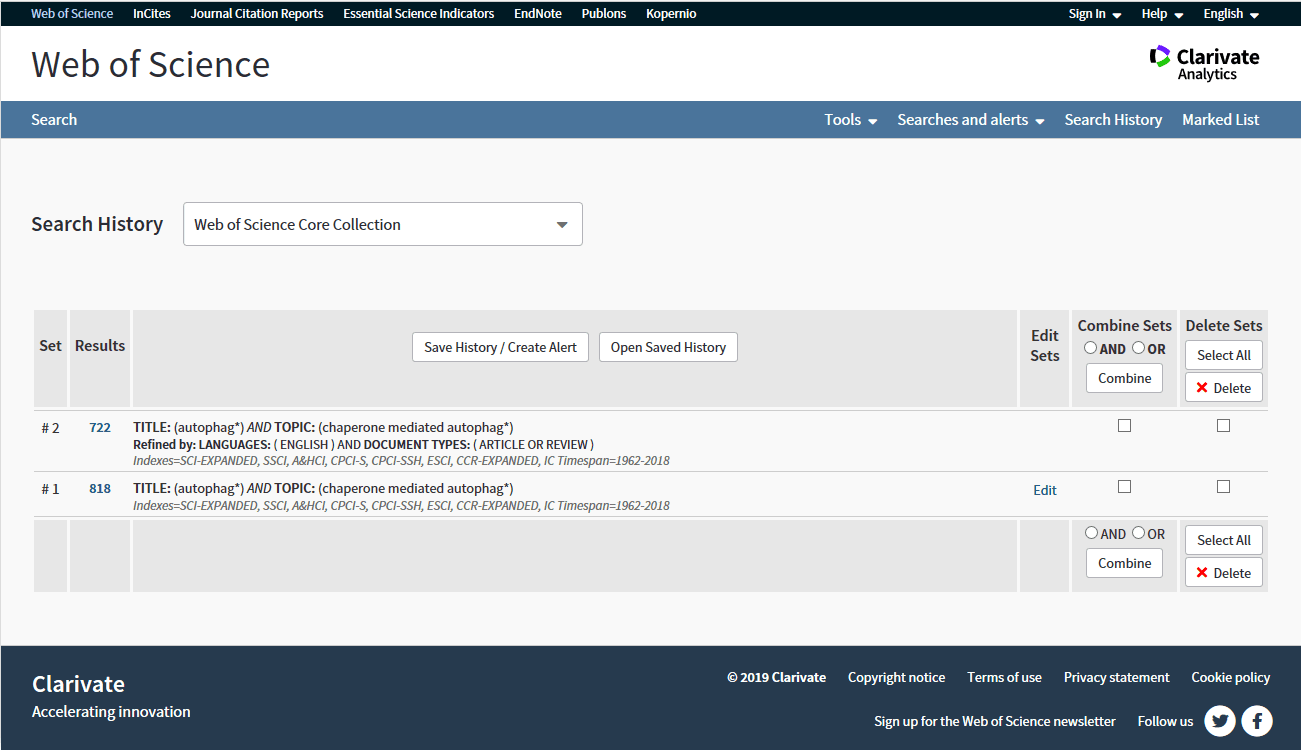

Supplement: Supplemental Information 2 [file peerj-07-7103-s002.zip › Search queries of chaperone medicated autophagy used in Web of Science Core Collection.png]

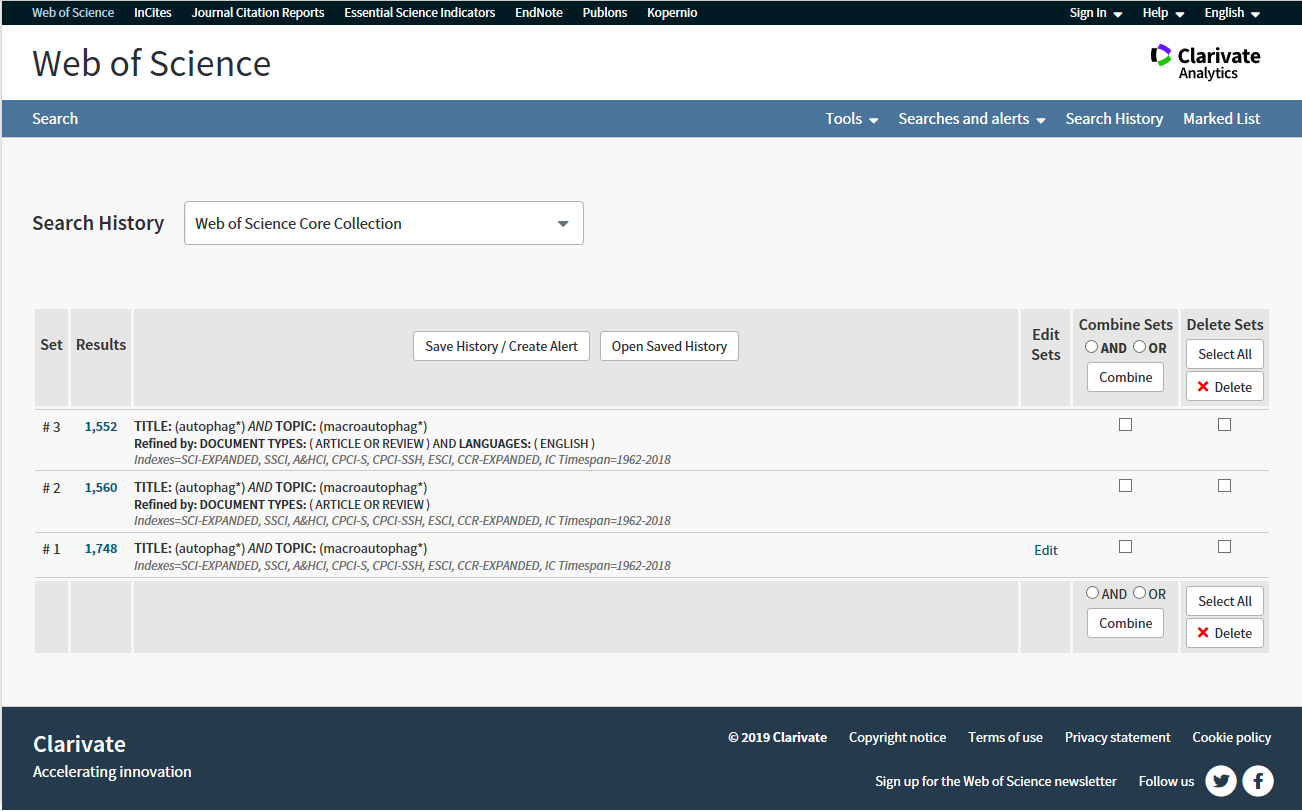

Supplement: Supplemental Information 2 [file peerj-07-7103-s002.zip › Search queries of macroautophagy used in Web of Science Core Collection.png]

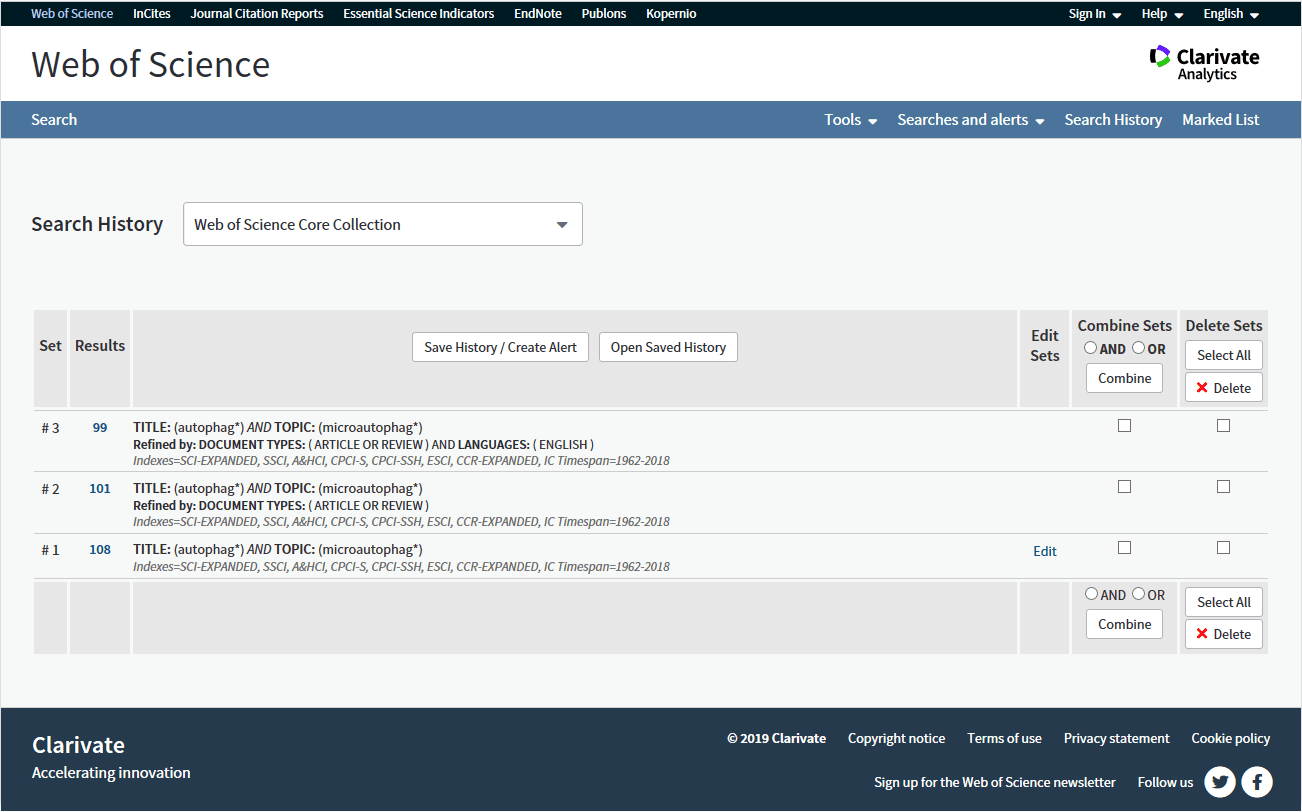

Supplement: Supplemental Information 2 [file peerj-07-7103-s002.zip › Search queries of microautophagy used in Web of Science Core Collection.png]

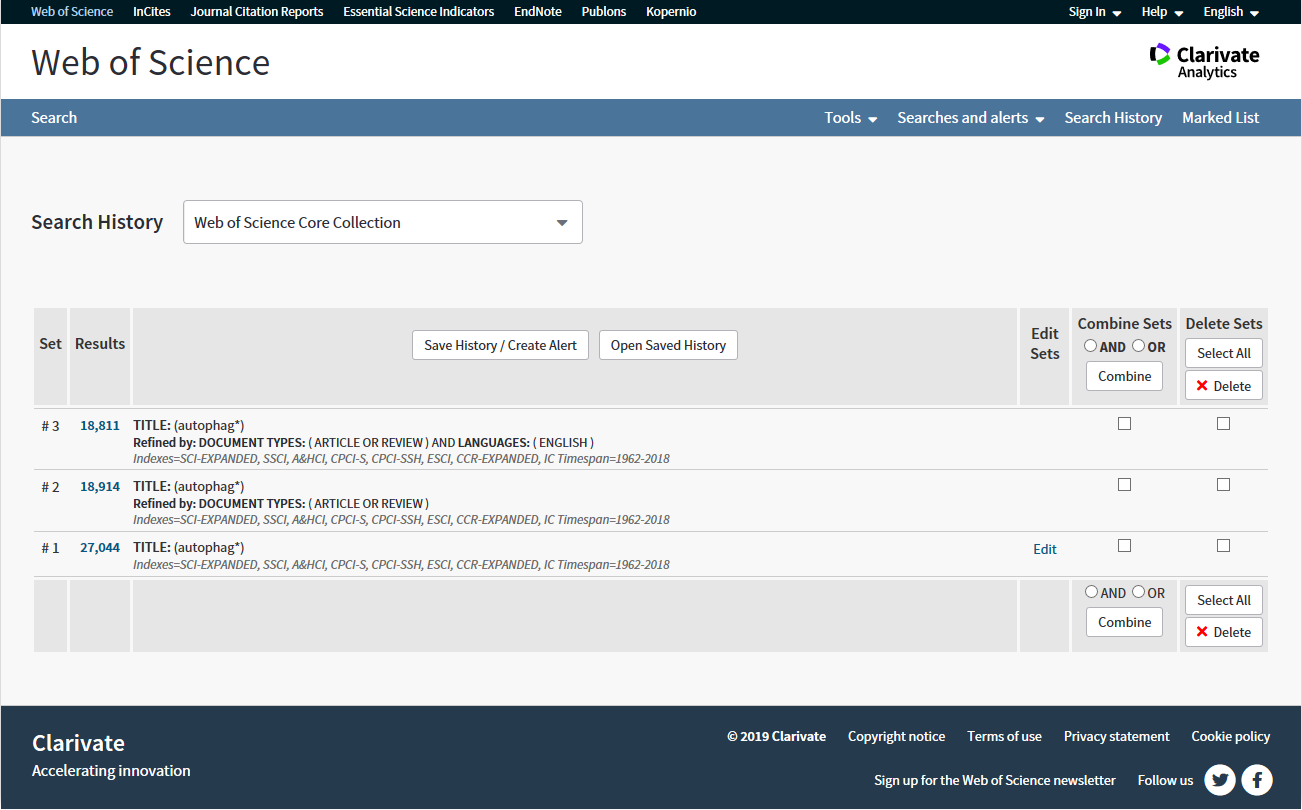

Supplement: Supplemental Information 4 [file peerj-07-7103-s004.png]

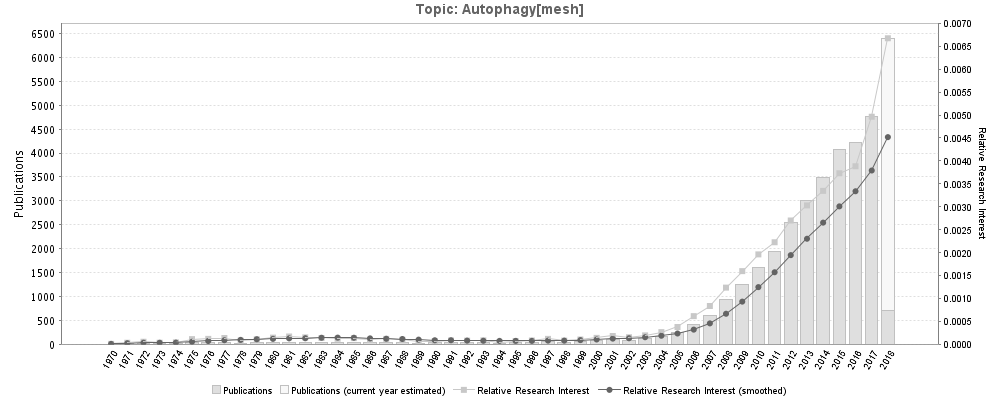

Supplement: Supplemental Information 5 [file peerj-07-7103-s005.png]

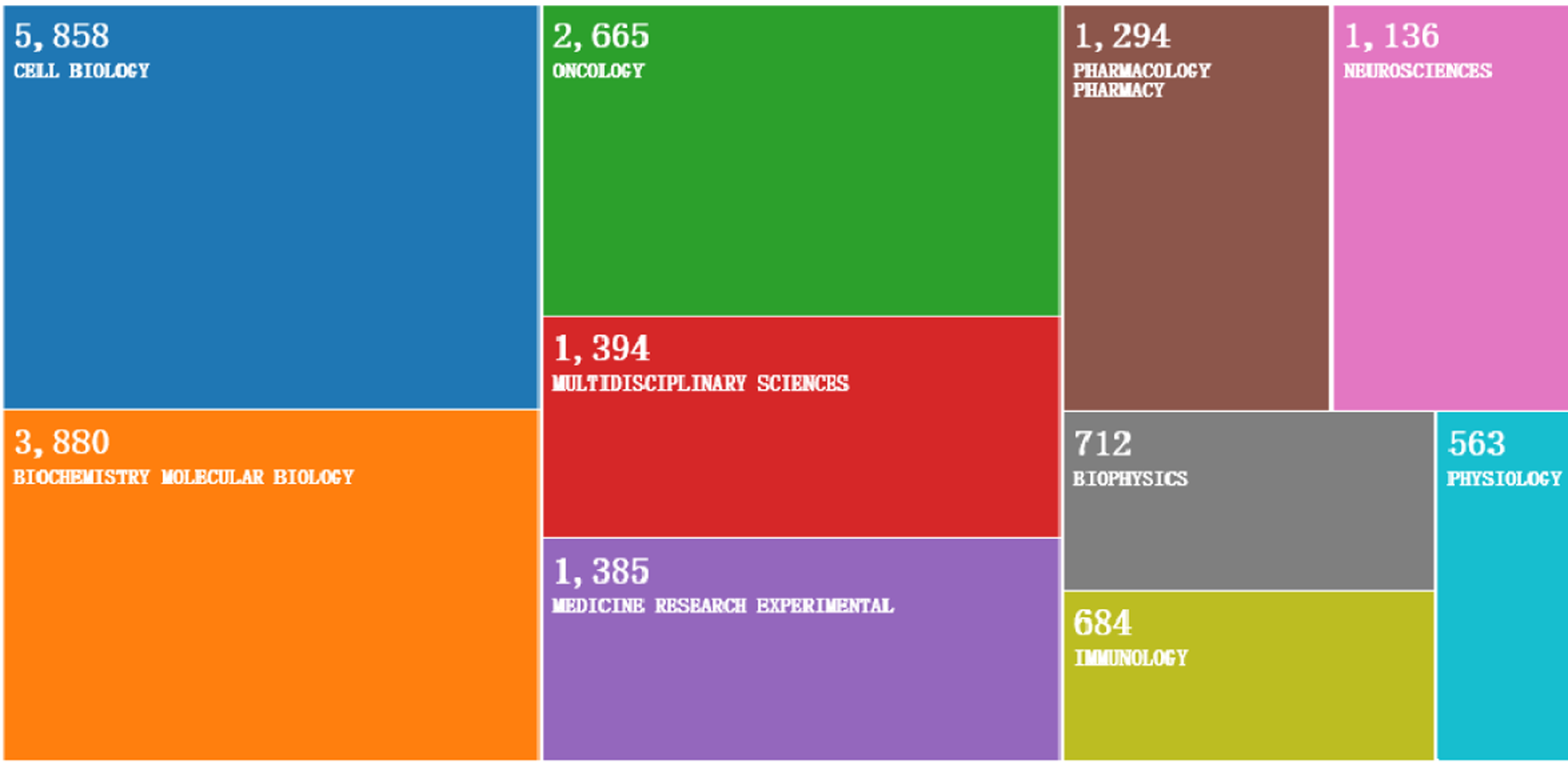

Supplement: Supplemental Information 6 [file peerj-07-7103-s006.png]

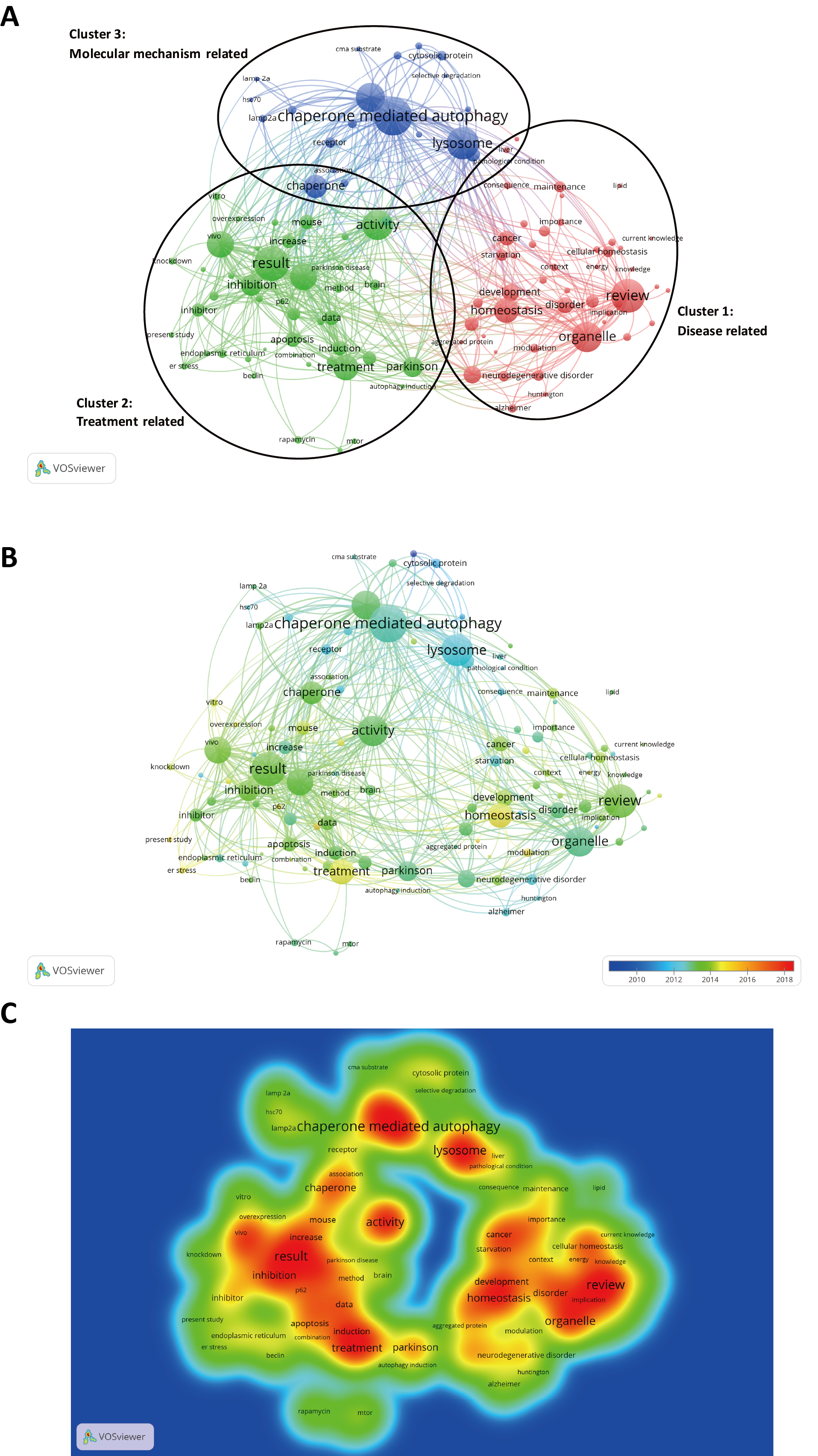

Supplement: Supplemental Information 7 [file peerj-07-7103-s007.png]

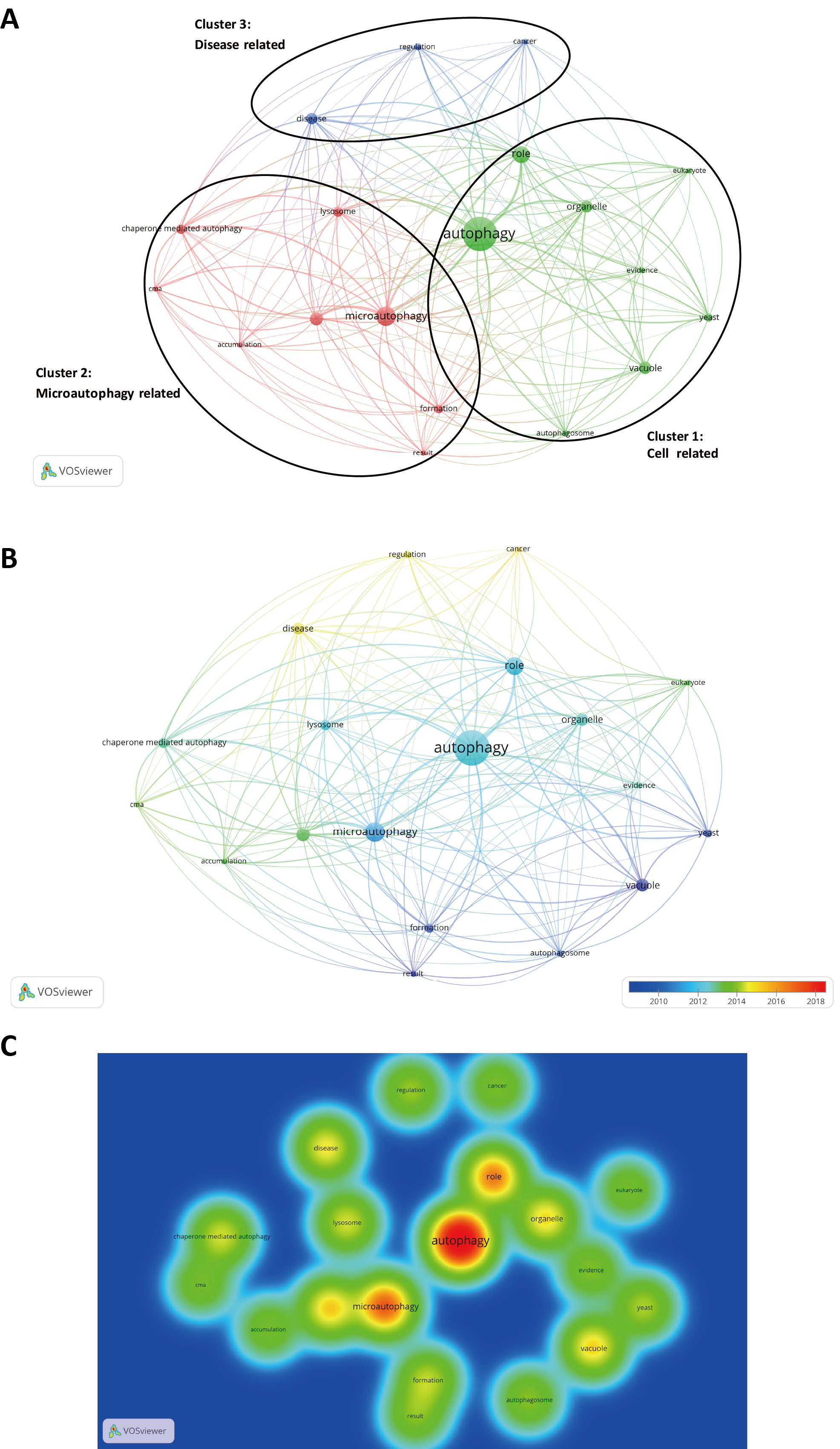

Supplement: Supplemental Information 8 [file peerj-07-7103-s008.png]
