# Supplementary material for: Bibliometric analysis of research on the trends in autophagy
Source: PeerJ. 2019 Jun 5;7:e7103. doi: 10.7717/peerj.7103 (PMC6556104; doi:10.7717/peerj.7103)
Supplement: Supplemental Information 3 [file peerj-07-7103-s003.docx]

Table S1 The H-index and citation frequency of top 10 productive authors on autophagy research

| Author | Number of Papers | Citation frequency | H index |
| --- | --- | --- | --- |
| Klionsky Daniel J | 171 | 38450 | 82 |
| Mizushima Noboru | 140 | 59826 | 90 |
| Ohsumi Yoshinori | 133 | 35640 | 77 |
| Yoshimori Tamotsu | 120 | 37018 | 70 |
| Cuervo Ana Maria | 116 | 26698 | 67 |
| Levine Beth | 111 | 46708 | 71 |
| Kroemer Guido | 108 | 28061 | 66 |
| Codogno Patrice | 102 | 16323 | 53 |
| Rubinsztein  David C | 102 | 23734 | 60 |
| Komatsu Masaaki | 98 | 25659 | 55 |
